# Effects of Omeprazole on Iron Absorption: Preliminary Study

**DOI:** 10.5152/tjh.2013.0042

**Published:** 2013-09-05

**Authors:** Mila Tempel, Anupama Chawla, Catherine Messina, Mahmut Yaşar Çeliker

**Affiliations:** 1 Department of Pediatrics, Stony Brook Long Island Children’s Hospital, Stony Brook, USA; 2 Department of Preventive Medicine, Stony Brook University Medical Center, Stony Brook, USA

**Keywords:** iron, Omeprazole, PPI, Proton pump inhibitors, Anemia

## Abstract

**Objective:** Increasing numbers of pediatric and adult patients are being treated with proton pump inhibitors (PPIs). PPIs are known to inhibit gastric acid secretion. Nonheme iron requires gastric acid for conversion to the ferrous form for absorption. Ninety percent of dietary and 100% of oral iron therapy is in the nonheme form. To the best of our knowledge, the effect of PPIs on iron absorption has not been studied in humans. Our study assessed the relationship between omeprazole therapy and iron absorption in healthy subjects.

**Materials and Methods:** We recruited 9 healthy volunteers between June 2010 and March 2011. Subjects with chronic illness, anemia, or use of PPI therapy were excluded. Serum iron concentrations were measured 1, 2, and 3 h after the ingestion of iron (control group). The measurements were repeated on a subsequent visit after 4 daily oral administrations of omeprazole at a dose of 40 mg (treatment group).

**Results:** One female and 8 male volunteers were enrolled in the study with a mean age of 33 years. There was no statistical difference detected between baseline, 1-h, 2-h, and 3-h iron levels between control and treatment groups.

**Conclusion:** Administration of omeprazole for a short duration does not affect absorption of orally administered iron in healthy individuals.

## Abstract (tr)

**Amaç:** Giderek artan sayıda pediatrik ve yetişkin hastalar proton pompa inhibitörleri (PPI) ile tedavi edilmektedir. PPI’ların mide asidini inhibe ettikleri bilinmektedir. Heme’e bağlı olmayan demir ferroz şekline geçerek absorb edilebilmesi için mide asidi gerektirir. Diyetteki demirin yüzde doksanı ve tedavide kullanılan demirin yüzde yüzü heme’e bağlı olmayan durumdadır. Bilgimize göre PPI’ların demir absobsiyonunda nasıl etki ettiği insanlarda araştırılmamıstır. Araştırmamız omeprazol tedavisi ile demir absorbsiyonu arasındaki ilişkiyi sağlıklı kişilerde incelemiştir.

**Gereç ve Yöntemler:** Haziran 2010 ile Mart 2011 arasında 9 sağlıklı gönüllüyü çalışmamiz için davet ettik. Kronik hastalığı veya anemisi olan kişilerle PPI tedavisinde olan kişileri dışladık. Serum demir konsantrasyonunu demir alımından 1, 2, ve 3 saat sonra ölçtük (kontrol grubu). Bu ölçümleri bir sonraki ziyarette 4 günlük oral 40 mg dozunda omeprazol tedavisinden sonra tekrarladık (tedavi grubu).

**Bulgular:** Ortalama yaşları 33 yıl olan 8 erkek ve 1 kadın gönüllü çalışmamıza katıldı. Kontrol grubu ile tedavi grubu arasında bazal, 1 saat, 2 saat, ve 3 saat sonraki demir konsantrasyonları arasında istatistiksel anlamlı bir fark görülmedi.

**Sonuç:** Sağlıklı kişilerde kısa bir zaman sürecince verilen omeprazol oral olarak alınan demirin absorbsiyonunu etkilemez.

## INTRODUCTION

Gastric acid plays an important role in the absorption of iron. Dietary iron can be divided into 2 types with respect to absorption: the heme type, derived from animal blood and muscle, which is well absorbed and comprises about 10%-30% of the normal Western diet, and the more common nonheme type, derived from plants (fruits, vegetables, grains, nuts), which requires an acidic gastric environment for absorption [1,2]. Although the heme component is absorbed independent of gastric pH, the nonheme part requires an acidic pH for absorption [3].

At physiologic pH and in the presence of oxygen, iron exists predominantly in the highly insoluble ferric Fe(III) form and therefore is poorly bioavailable [4]. Ingested ferrous ions are readily oxidized to ferric ions by dissolved oxygen [5]. Stomach acid is important in releasing iron from ligands in food and in solubilizing ferric iron by converting it to ferrous form [6].

It has been speculated that inhibition of gastric acid secretion may lead to reduction in iron and vitamin B12 absorption [7]. The development of iron malabsorption has been shown in a variety of hypo- or achlorhydric conditions. Heath and Patek observed that among patients with iron-deficiency anemia, the rate of the hemoglobin response to iron therapy was lower in those who had achlorhydria than in those without [8]. Goldberg et al., using radiolabeled iron, showed that patients with achlorhydria have diminished absorption of iron [9]. These data clearly demonstrate the importance of gastric acid secretion in iron absorption.

Increasing numbers of pediatric and adult patients are being treated with proton pump inhibitors (PPIs), often for several months. There is a limited amount of research specifically looking at the effects of PPIs and absorption of orally administered iron. Our study is the first such human study to assess this relationship in healthy subjects.

## MATERIALS AND METHODS

The protocol was approved by the institutional review board and written informed consent was obtained from all subjects. Subjects were recruited from a pool of healthy volunteers, 18 to 50 years old, who responded to our advertisement. Those with low ferritin levels <10 ng/mL for females, <20 ng/mL for males) or anemia (hemoglobin <115 g/L for females, <130 g/L for males), pregnant, or with malabsorption disorders, major gastrointestinal surgery, malignancy or any chronic conditions involving the gastrointestinal system, renal disease, or hematological, cardiac, or pulmonary conditions were excluded during the screening visit. Menstruating females came to their study visits 10-13 days after their last day of menstrual bleeding in order to synchronize menstrual cycles at the time of testing. All subjects were asked to continue their regular diet as we intended this study to be applicable to real-life situations.

At the second visit, baseline serum iron level and total iron-binding capacity were obtained after a minimum of 8 h of fasting. Ferrous sulfate (650 mg; 130 mg of elemental iron) was then administered orally as a tablet preparation, followed by determination of serum iron concentration 1, 2, and 3 h after the ingestion of iron. Male subjects returned 1 week and female subjects returned 1 month later for their last visit. Subjects were instructed to take omeprazole (40 mg) daily 30 min before breakfast for 4 days prior to their third visit (post-PPI), when after a minimum of 8 h of fasting, subjects were given 40 mg of omeprazole. During this visit, 650 mg of ferrous sulfate was administered orally. Serum iron concentration was measured before and 1, 2, and 3 h after the ingestion of iron. Baseline and post-omeprazole serum iron level determination was done around the same time of the day at each visit.

## RESULTS

We recruited 9 healthy volunteers, 8 males and 1 female, who completed the study between June 2010 and March 2011. Mean age at the time of study was 33. A single blood sample in 2 subjects was hemolyzed and therefore excluded from the analysis.

**Figure 1:**
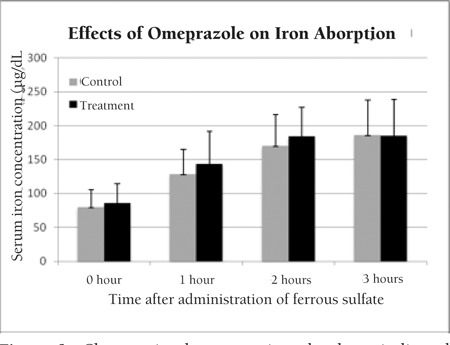
Changes in the serum iron levels at indicated time points after single parenteral administration of ferrous sulfate at 6 mg elemental iron/kg per dose (max. 130 mg of elemental iron) compared to baseline (hour 0).

Serum iron levels measured before taking omeprazole (control) and after 5 days of omeprazole therapy (treatment) were compared (Figure 1). When serum iron concentrations at different time points were compared between the control and treatment groups, there was no statistical difference (Table 1).

## DISCUSSION

After oral administration of PPI, the onset of the antisecretory effect of omeprazole occurs within 1 h, with the maximum effect occurring within 2 h [10]. The inhibitory effect of omeprazole on acid secretion increases with repeated once-daily dosing, reaching a plateau after 4 days. Bastani et al. showed a significant rise in serum iron concentration of 164±32% µg/dL above baseline 2 h after oral ingestion, plateauing afterwards [11]. These studies suggested that a 5-day course of PPI treatment would be sufficient length of treatment and 3 h of serum iron-level monitoring would be sufficient to detect serum changes in iron levels following the ingestion of iron preparation. 

Our study was done in healthy subjects to minimize factors other than PPIs that may influence iron absorption. We did not restrict the diet of the subjects to maintain real-life applicability. In rats that were on a normal diet, omeprazole did not alter iron absorption, in agreement with our results [12]. 

Previous human and animal studies suggested that omeprazole may inhibit iron absorption in iron-depleted states. Two adult patients with iron deficiency anemia from erosive gastritis had their anemia corrected only after discontinuation of omeprazole [13]. Omeprazole inhibits iron absorption in rats that were fed an iron-deficient diet [12]. Another study showed that treatment of patients with hereditary hemochromatosis with PPIs led to significant reduction in the volume of blood removed annually [14]. In that report, postprandial iron absorption studies in 14 subjects with hemochromatosis demonstrated decreased absorption of nonheme iron from a test meal by approximately 50% after 7 days of PPI therapy. These studies suggest that PPIs may inhibit iron absorption in iron-depleted as well as iron-overloaded individuals.

Although our sample size was small, it was calculated based on prior studies in which serum iron levels decreased by 50% after 7 days or more of PPI therapy in hemochromatosis patients and a case report by Sharma et al. [13,14]. Ad hoc sample size calculations using iron levels from the 3 h following treatment showed that we would have needed 117,268 subjects in each group for the observed difference to be statistically significant. This suggests that the small sample size is not likely to account for our observations.

## CONCLUSION

Our study demonstrates that short duration of omeprazole use does not affect iron absorption in iron-replete healthy individuals who are on a normal diet. Since omeprazole’s effect was shown in iron-depletion or iron-overload states, it is plausible that omeprazole may have an effect on iron absorption only in abnormal iron metabolic states. Although short-term omeprazole therapy does not appear to affect iron absorption, since PPIs are increasingly being used for longer durations, further studies are warranted to evaluate their effects on long-term use. 

## COMPETING INTERESTS

There are no financial or nonfinancial competing interests to disclose by any of the authors.

## Figures and Tables

**Table 1:**
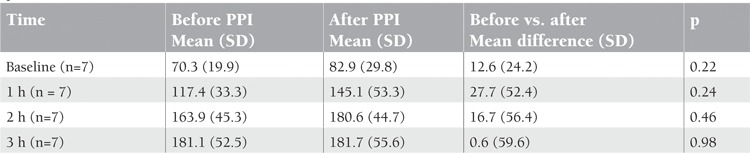
Mean change in serum iron concentrations (μg/dL) in subjects before and after oral iron challenge (paired samplest-test). SD: standard deviation
